# Interference-Aware Adaptive Beam Alignment for Hyper-Dense IEEE 802.11ax Internet-of-Things Networks

**DOI:** 10.3390/s18103364

**Published:** 2018-10-09

**Authors:** Dohyun Kwon, Sang-Wook Kim, Joongheon Kim, Aziz Mohaisen

**Affiliations:** 1 School of Computer Science and Engineering, Chung-Ang University, Seoul 06974, Korea; kdh1102@cau.ac.kr (D.K.); swkim4@cau.ac.kr (S.-W.K.); 2 Department of Computer Science, University of Central Florida, Orlando, FL 32816, USA

**Keywords:** IEEE 802.11ax, multiple NAVs, Internet-of-Things (IoT), beamforming

## Abstract

The increasing use of Internet of Things (IoT) devices in specific areas results in an interference among them and the quality of communications can be severely degraded. To deal with this interference issue, the IEEE 802.11ax standard has been established in hyper-dense wireless networking systems. The 802.11ax adopts a new candidate technology that is called multiple network allocation vector in order to mitigate the interference problem. In this paper, we point out a potential problem in multiple network allocation vector which can cause delays to communication among IoT devices in hyper-dense wireless networks. Furthermore, this paper introduces an adaptive beam alignment algorithm for interference resolution, and analyzes the potential delays of communications among IoT devices under interference conditions. Finally, we simulate our proposed algorithm in densely deployed environments and show that the interference can be mitigated and the IEEE 802.11ax-based IoT devices can utilize air interface more fairly compared to conventional IEEE 802.11 distributed coordination function.

## 1. Introduction

**Figure 1:**
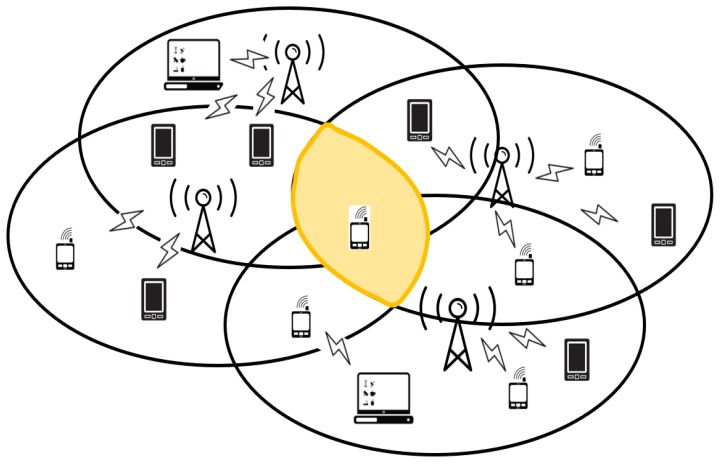
Scenario of hyper-dense APs: multiple inter-BSS NAVs may deteriorate medium utility chance of STA in OBSS.

According to a recent Cisco technical report [1], there will be 3.5 networked devices per capita by 2021, from 2.3 networked devices per capita in 2016. The trend of the ever-increasing number of mobile devices led to a tremendous deployment of access points (APs) for increasing user satisfaction. As a result, congestion and contention problems among devices in overlapping basic service set (OBSS)—which severely deteriorates the user experience, as shown in Figure 1, become important. For efficiently handling this issue, the task group of IEEE 802.11ax (TGax) is conducting IEEE 802.11ax standardization, in what is called the high-efficiency wireless local area networks (HE-WLAN) fundamentally based on IEEE 802.11ac to address the congestion problems [2,3,4,5].

The 802.11ax HE-WLAN adopts a new scheme, called the multiple network allocation vectors (NAVs), in order to mitigate the interference problem. Each IEEE 802.11ax-based Internet-of-Things (IoT) station holds multiple NAVs to avoid wireless congestion. If an STA is located in a certain area which OBSS created by three APs, the STA holds three different NAVs for the corresponding APs. Specifically, the NAV that is related to the associated AP is called *intra-BSS NAV*, whereas the two other NAVs are called *inter-BSS NAV*. The intra-BSS NAV and inter-BSS NAV are utilized for avoiding interference among STAs, and are located within the associated AP and other alien APs, respectively. If the STAs are associated with the AP, then the intra-BSS NAV is set and the STA should be idle until the reception of *contention free end frame* from the AP. On the other hand, if STAs that are associated with other alien APs are communicating among themselves, the STA should be idle due to the inter-BSS NAV. That is, multiple NAVs consist of intra-BSS NAV and inter-BSS NAVs. In addition, the multiple NAVs behave strategically to avoid interference among STAs in OBSS networks and can be utilized properly to transmit data for each STA. To sum up, if at least one of inter-BSS NAVs or intra-BSS NAV is set in a specific subcarrier the STA cannot transmit data because it assumes the channel is busy. Envisioning a future WLAN which configures hyper-dense deployment of countless devices in a certain small area, STA may set multiple inter-BSS NAVs and could consequently be excessively delayed for even single transmission.

To avoid excessive delay, we propose a novel algorithm to address the delay problem of STA with data in its data queue that needs to be transmitted and is blocked due to several inter-BSS NAVs or intra-BSS NAV settings. This means that the multiple NAVs can be helpful for avoiding interference problem but it can aggravate delay. Therefore, this paper targets a new objective, i.e., a joint optimization of delay reduction and interference minimization. If the time interval of the idle state exceeds a threshold, the delayed STA decides with which AP to be associated among its own AP and other alien APs, and swiftly finds a direction towards the candidate AP within a low time complexity. Finally, the STA configures a beamformed-beam to transmit data to the designated AP in the calculated direction. Our algorithm in IEEE 802.11ax enables an STA to transmit data in a densely deployed environment even though it has a set of inter-BSS NAVs or an intra-BSS NAV. We demonstrate an analytical model of the proposed algorithm and simulate its performance compared to IEEE 802.11 distributed coordination function (DCF) in terms of fairness and expected transmission time loss.

The contribution of this paper is an algorithm which mitigates the delay issue of STAs in a heavily dense deployment scenario of 802.11ax-based IoT wireless networks. For this purpose, we propose a low-complexity and swift beam direction selection algorithm that is based on opportunistic beamforming MAC protocols.

The rest of this paper is constituted as follows. Section 2 introduces related work and the enhanced features of IEEE 802.11ax are summarized in Section 3. Section 4 proposes an opportunistic beamforming algorithm and describes the analytic model of the proposed algorithm. Section 5 performs intensive simulations and presents discussions based on the simulation results. Finally, Section 6 concludes this paper.

## 2. Related Work

**Figure 2:**
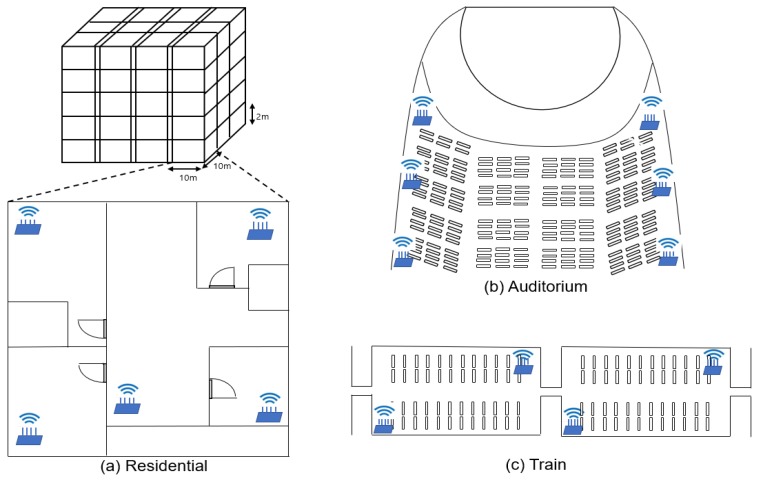
Three densely deployed APs scenarios of wireless devices. (**a**) represents a plausible residential scenario which is composed of densely deployed multiple APs in an apartment. (**b**) is a scenario for multiple APs in an auditorium. (**c**) is an example of densely installed APs scenario in a carriage.

The dense deployment of IoT devices in the aforementioned scenario can be represented as in Figure 2. IEEE 802.11ax is designed for high performance distributed networking in dense deployment scenarios, such as carriages, residential apartments and auditoriums. Based on the nature of IEEE 802.11ax, it is one of the most suitable solutions for hyper-dense IoT wireless communications. By adopting the aforementioned multiple NAVs strategy, data transmission in dense deployment scenarios where tens of APs and hundreds of STAs co-exist simultaneously in a small area can be delayed due to wireless medium resource competition. That is, we can easily envision that each device should wait until all NAVs are reset when there exists multiple IoT devices that want to aggressively utilize the air interface. In this section, previous research results and primary features of the 802.11ax standard are discussed.

The research results in [6] demonstrated a downlink multi-user MIMO (DL MU-MIMO) along with new features of IEEE 802.11ax, i.e., uplink MU-MIMO (UL MU-MIMO) transmission in AP-initiated scenarios. In [6], the differences between legacy IEEE 802.11 wireless local area networks (WLANs) and IEEE 802.11ax were introduced. In addition, the analytical saturation throughput model of transmission scenarios for both single-user (SU) and multi-user (MU) was proposed. However, the authors did not consider realistic experiments, such as miscellaneous traffic (non-saturation case) between AP and multiple STAs. Meanwhile, the IEEE 802.11ax and IEEE 802.11ac were compared in [7]. The insights of DL/UL orthogonal frequency division multiple access (OFDMA), dynamic clear channel assessment (CCA), and UL MU-MIMO concepts were also introduced in [7]. Surveys on OFDMA-based medium access protocols and OFDMA-based concurrent MU medium access control algorithms for IEEE 802.11ax were presented in [8,9]. The authors elaborated various aspects of the next generation WLAN standard, 802.11ax, with respect to MU transmission, quality of service (QoS) guaranteed scheduling (resource allocation), and low signaling overhead. However, they were limited to compare several previously conducted works, and novel and realistic experiments were not proposed.

The research results in [10,11] were focused on dynamically tuning of CCA which enhances spatial reusability for addressing OBSS congestion problems. In terms of implementation, the link-system level simulator for IEEE 802.11ax with network simulator 3 (NS3) was explored in [12]. In [12], plausible transmission parameters, dense deployment scenarios, and the simulated throughput of each BSS with various numbers of STAs were studied. The authors of [13] studied QoS support in legacy IEEE 802.11 wireless networks and summarized the IEEE 802.11ax standardization processes. In addition, they presented an overview of current and expected features of IEEE 802.11ax in terms of the medium access control. Moreover, emerging long-term evolution licensed-assisted access which satisfied user needs with low latency and high bandwidth were jointly considered with IEEE 802.11ax for collaboration between cellular networks and IEEE 802.11 WLAN-based wireless networks. Besides, the method in [14] suggested a next generation medium access control mechanism in unlicensed bands (5G-U) for vehicular radio access with licensed-assisted access (LAA) and IEEE 802.11ax wireless networks. A new medium access control algorithm under resource uncertainty and physical sidelink shared channel (PSSCH) was proposed for LTE vehicle-to-everything (V2X), channel pre-occupation, and resource binding strategies. The algorithm in [15] proposed co-existence mechanisms of IEEE 802.11ax with cellular, LTE with unlicensed (LTE-U), and LAA. The authors of [15] considered single unlicensed frequency band transmissions where the locations of Wi-Fi STAs, Wi-Fi APs, and LTE evolved node B (eNB) are modeled as three independent homogeneous Poisson point processes. They derived analytical models for a set of metrics including signal-to-interference-plus-noise ratio coverage probability, density of successful transmissions, and Shannon throughput probability for both UL and DL of IEEE 802.11ax wireless and cellular networks.

There exist several improvements in IEEE 802.11ax for various purposes to enhance data rate in order to mitigate interference and improve the efficiency of frequency utilization. Among them, target wake time (TWT) is one of the improvements in IEEE 802.11ax. The TWT has advantage in terms of collision probability reduction in high-dense distributed IEEE 802.11-based WLAN network scenarios with pre-knowledge awake time of STAs with associated AP. The mechanism of TWT is extremely simple yet effective by calculating and analyzing when STA wakes up to transmit data over time. Furthermore, the TWT enables STAs to operate energy efficiently with the doze mode. For example, the authors of [16] proposed a scheduling scheme in IEEE 802.11ax with TWT to efficiently handle MU transmissions and multi-AP cooperation. The authors in [17] explored several new IEEE 802.11ax UL scheduling mechanisms and compared them among the maximum throughput of MU. They conducted evaluations of MIMO and OFDMA transmissions in IEEE 802.11ax against the carrier-sense multiple access with collision avoidance (CSMA/CA) MAC of IEEE 802.11ac with the SU and MU modes for various numbers of STAs scenarios in both reliable and unreliable channel environments. The authors of [18] suggested advanced IEEE 802.11ax TCP-aware scheduling strategies to optimize the performance using MU UL and MU DL transmissions. They were based on transmission opportunities (TXOP) and can control achieved goodput versus delays. The authors showed that the minimal goodput degradation strategy can reduce delay. In [19], the authors proposed a semi-matching-based load balancing scheme for hyper-dense IEEE 802.11 wireless networks. The scheme is operated in a centralized controller which makes decision whether the load is unevenly distributed among APs and controls the overall network throughput to be maximized. By taking advantage of IEEE 802.11ax new feature considerations, simultaneous transmit and receive (STR) which is also called full-duplex, is likely to be applied in legacy IEEE 802.11-based wireless standards. One of the key challenges is to integrate the STR mode with minimal modifications into legacy standards. In [20], the authors proposed a simple yet practical approach to enable the STR mode in IEEE 802.11-based wireless networks with co-existing full-duplex and half-duplex STAs. TGax for IEEE 802.11ax standardization plans to adopt some new features such as dynamic CCA tuning, multiple NAVs, and TWT. The TWT is an energy efficient strategy in terms of the operation of STA considering that the associated AP of the STA has full knowledge of awake schedule of the STA and thus the AP properly transmits data by calculating the awake time of STA which is not in doze mode. In addition, a multi-NAVs strategy for efficient frequency utilization is considered for mitigating wireless interference in hyper-dense situations. The STA set NAV for an AP where the AP initiates transmission. If at least one of the inter-BSS NAVs or intra-BSS NAV of STA is set, the STA should be idle for avoiding congestion until all NAVs are reset. In the following subsections, IEEE 802.11ax specific wireless frame format and the error correction and modulation of IEEE 802.11ax are proposed.

## 3. IEEE 802.11ax Features

There are several newly proposed candidate features in the 802.11ax: dense constellation (1024 quadrature amplitude modulation (QAM)) for higher data rate, interference mitigating multiple NAVs, energy efficient operation of STAs with TWT, wider range of wireless signal transmission and reception compared to legacy standards (802.11ac) with standards-based sounding and beamforming technology. Beside these candidates, fundamental key features of 802.11ax concerning the physical layer frame format, error correction and modulation strategy are introduced. These new features are highly related and are used for enabling communication between STAs in 802.11ax WLAN, and so they affect interference among STAs. In the following subsection, the aforementioned features of 802.11ax are introduced in more details.

### 3.1. PPDU Frame Structure

**Figure 3:**
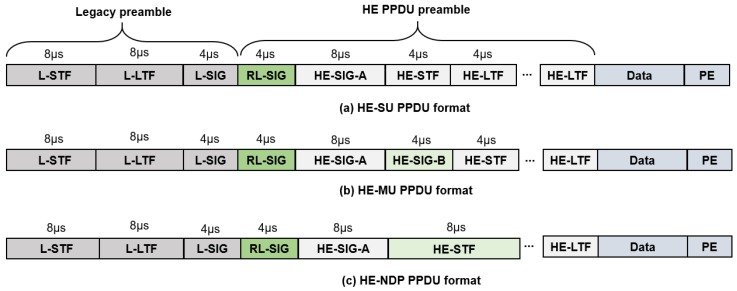
IEEE 802.11ax PPDU frame format.

IEEE 802.11ax TGax standard changed the physical protocol data unit (PPDU) frame format compared to IEEE 802.11ac for improving frequency, channel, and medium utilizations. As shown in Figure 3, the IEEE 802.11ax PPDU frame formats keep the legacy preambles for backward compatibility and add *RL-SIG* field behind the legacy preambles for automatic detection of IEEE 802.11ax frames. In addition, IEEE 802.11ax IoT devices detect channel state information (CSI) by referring to the *L-LTF* field in Figure 3. If the result of modulo three operations of the length field of *L-SIG* is 1, the frame is for SU. If the result is 2, then the frame is for MU transmission. A null data packet (NDP) frame for CSI exchange is in Figure 3c and is transmitted by AP. When an STA receives NDP announce (NDPA) frame followed by NDP, the STA transmits CSI report frame to AP and the AP transmits trigger frame as a response to CSI report to initiate UL transmission.

### 3.2. Error Correction and Modulation

The legacy IEEE 802.11 standard adopted block check character (BCC) for forward error correction (FEC), and not the low-density parity check (LDPC) due to its high computational cost. However, IEEE 802.11ax selects LDPC for error correction as mandatory because it performs better in terms of capacity compared to IEEE 802.11ac. In addition, IEEE 802.11ax selectively utilizes dense constellation, such as 1024 QAM, so that each symbol contains more information and achieves an improved throughput performance. Furthermore, IEEE 802.11ax exploits four-fold larger fast Fourier transform (FFT) size compared to the IEEE 802.11ac so that the spectral efficiency can be improved for appropriate dense scenarios, as shown in Table 1. Although its subcarrier is narrower than before, the inter-symbol interference (ISI) problem is relieved by setting longer guard interval (GI). By utilizing these improvements in terms of medium access control, data transmission under severe delay spread in outdoor environments can be successful.

## 4. Proposed Opportunistic Medium Access

**Figure 4:**
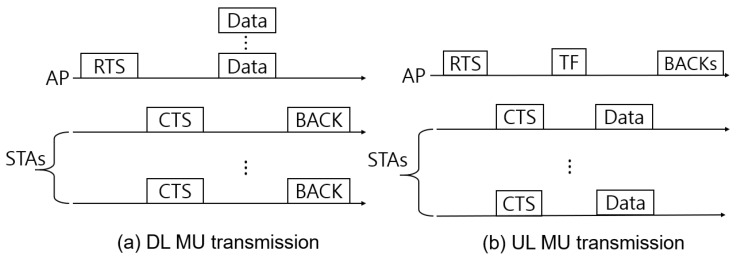
Simplified DL and UL transmissions of IEEE 802.11ax.

In this section, we discuss an excessively long delay problem that may occur in IEEE 802.11ax and propose a swift and low-complexity beam direction selection algorithm based on opportunistic beamforming medium access control. First, the simplified mechanism of the DL and UL transmissions of IEEE 802.11ax is as illustrated in Figure 4. In case of DL MU transmission, the data packets from an AP are transmitted simultaneously to multiple STAs and each STA replies the corresponding *block* ACK to the associated AP. On the other hand, STAs, which have packets to transmit simultaneously, send their packets as they receive the *trigger frame*. In the following subsections, the mechanism and analytical model of the proposed medium access control algorithms and related assumptions are presented.

In terms of the various evaluation criteria used for our algorithms and previous research, and used for comparison between the previous research [21,22,23,24] and our algorithm is proposed as Table 2. The MU transmission and MU access represent feature of simultaneous channel access and concurrent data transmission of STAs by taking advantage of the frequency orthogonality of OFDMA. Hence, channel access efficiency of STAs can be enhanced by utilizing this feature. In addition, MU diversity stands for the characteristic that each STAs can be allocated only high channel gain resource blocks (RBs) and can improve their throughput performance. Simple signal exchange shows that simple control message exchange procedure in communication system. In case of 802.11ax and our proposed protocol, trigger frame, which is one of the control messages among data transmission procedure, is related to this feature. Finally, channel state information (CSI) measurement item refers to the measurement process of channel state between AP and STAs. The AP and STAs exchange the result of this procedure and utilize the information of channel to appropriately tune their transmission parameters.

### 4.1. System Model and Assumptions

**Figure 5:**
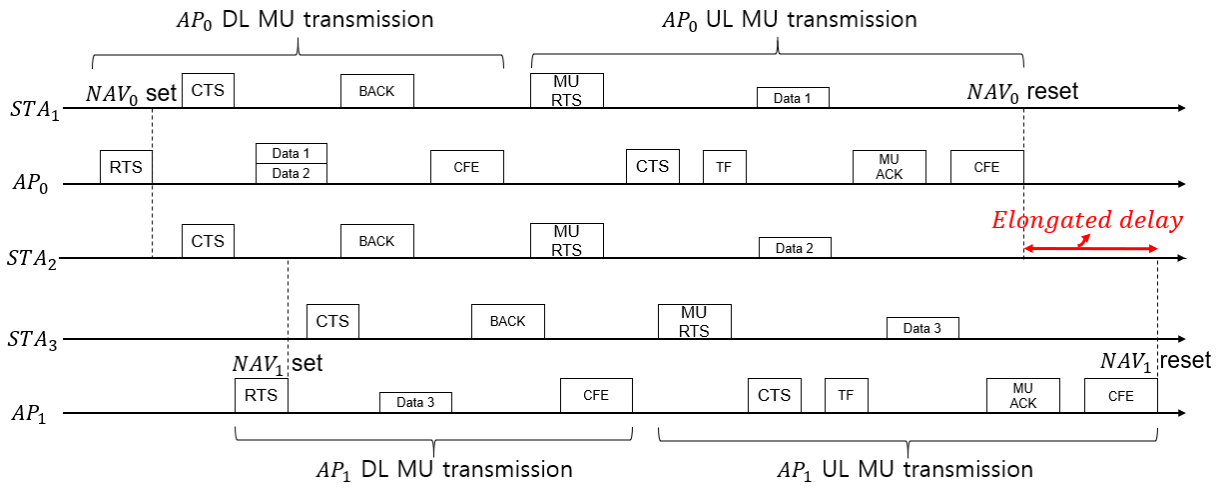
Elongated delay scenario of $STA_{2}$ due to inter-BSS NAVs $NAV_{1}$ setting.

In order to understand our proposed algorithm, consider a simple IEEE 802.11ax network which consists of multiple OBSSes with five APs and twenty STAs in a narrow area as presented in Figure 5 (only two APs and five STAs are represented for simplification). In particular, the user experience of STA${}_{2}$, which is our main focus, is deteriorated during the red-lined duration in Figure 5 due to multiple inter-BSS NAVs setting. It is straightforward to envision that the red-lined duration will get longer while the more affected APs are increased. Thus, this phenomenon can be much more severe and the STA hardly transmits data due to increased delays in hyper-dense wireless networking scenarios. If affecting alien APs are relaying to transmit for each transmission cycle, it is obvious that the STA gets the worst chance to transmit data. To address this issue, we propose a low-complexity and swift beam direction selection algorithm based on *opportunistic beamforming* data transmission for letting delayed STA transmit data. Therefore, the proposed algorithm consists of two modules: (i) appropriate beamformee designation and direction configuration procedure and (ii) data transmission through the direction with beamforming.

In order to analyze our proposed algorithm in hyper-dense IEEE 802.11ax wireless networks, the following assumptions are made.

- Each STA has exactly the same performances with OFDMA and MU-MIMO for mathematical performance analysis.
- In addition, antennas which are installed in each IEEE 802.11ax IoT device are full-duplex vouching simultaneous U/DL transmission.
- Detailed parameter setting of antennas for beamforming including azimuth, half power beam width (HPBW), and antenna gains are not associated with the proposed algorithm.
- U/DL MU transmissions are considered in this paper.
- In our proposed algorithm, NAV is set to 0 if the corresponding AP is idle and vice versa.

### 4.2. Beam Direction Selection and Beamforming Algorithm

**Figure 6:**
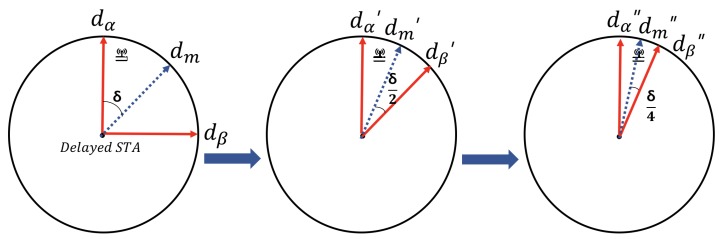
Detailed mechanism of $Search_{HR}^{e}\left(\langle d_{\alpha },d_{\beta }\rangle ,BSR\right)$ in Algorithm 1.

This section introduces a fast and lightweight beam direction selection-based beamforming algorithms to address the aforementioned issue in IEEE 802.11ax wireless networks. (i) If an STA attempts to transmit data, it considers if its inter-BSS NAVs are set. If all of them are reset then it immediately transmits data to its associated AP (line 14 of Algorithm 1). However, if at least one of them is set, the STA should not act selfish and transmit data to avoid inter-BSS congestion deterioration. In addition, the delayed STA due to inter-BSS NAV setting investigates a new AP to associate after a specific duration $\theta_{ed}$, i.e., the STA has wait threshold variable ω to avoid excessive delays and only finds the appropriate direction of beamformee after the ω exceeds the waiting threshold $\theta_{ed}$ (line 3 of Algorithm 1). (ii) After the STA makes decision to find the beamformee, it chooses the associated AP or alien APs by sorting them based on a descending order of their signal strengths (line 2 of Algorithm 2) and finds the most appropriate direction (line 3 to 13 of Algorithm 2). (iii) STA beamforms towards its beamformee in the direction of dir, which is the result of Algorithm 1 (line 7 to 11 of Algorithm 1). Notice that the beam alignment procedure is recursively called as in Figure 6 for finding the suitable beam direction angle (azimuth and elevation angle of beamforming) between the delayed STA and target AP which is within the range of the HPBW of STA. The STA actively searches with a beam search rate (BSR) between the originally associated AP or other alien APs based on the received signal strength indicators (RSSI) of each AP, where the wireless data traffic loads on each AP are closely similar so that the RSSI is a proper criterion for selecting the AP. Based on our assumptions, if the STA chooses to transmit data via its associated $AP^{a}$, it utilizes the corresponding information which already has, and exploits it for searching direction set of APs in low-resolution ($LR_{dir}^{a}$) within ($AP_{CS}^{a}$) direction set under BSR (line 4 of Algorithm 2). After that, the STA searches a beamformee with a higher resolution ($HR_{dir}^{a}$) by exploiting the result of low-resolution direction (line 5 of Algorithm 2). The main difference between low resolution and high resolution is the searching direction size in a circular sector (CS) scale, which is the range of direction searching procedure where low resolution is wider than the high resolution’s one. On the other hand, if the STA chooses to transmit among one of the alien $AP^{e}s$, the STA sorts them considering the signal strength with a descending order and selects one of them (line 8 to 9 of Algorithm 2). Later, the STA sets CSI between $d_{\alpha }$ and $d_{\beta }$, which is the initial range of searching direction area (line 10 of Algorithm 1), and finds the direction of targeted alien AP ($HR_{dir}^{e}$) by calling $Search_{HR}^{e}$ function recursively while dynamically tuning the $d_{\alpha }$ and $d_{\beta }$ per every function call (line 11 of Algorithm 2). The detailed operation of $Search_{HR}^{e}\left(\langle d_{\alpha },d_{\beta }\rangle ,BSR\right)$ is represented in Figure 6. After these processes, the STA finally sets up CSI between the selected $AP^{e}$ and gets the joint beamforming information (line 12 to 13 of Algorithm 2). Finally, it can be observed that the STA addresses delay issue and the STA can transmit data to either $AP^{a}$ or $AP^{e}$ in the direction of the calculated result in order to improve the performance in terms of the interference reduction. To sum up, the STA with our proposed beamforming algorithm in hyper-dense environments does not act selfishly but little tenacious to cooperate with other STAs in OBSS and to avoid wireless congestion. In addition, as illustrated in Figure 6, if the STA makes a decision to associate with $AP^{e}s$, then finding a dominant $AP^{e}$ and searching appropriate beam direction are required with the complexity of $O\left(n\log(n)\right)$, according to the fact that finding dominant $AP^{e}$ among $AP^{e}s$ requires $O\left(n\right)$ and searching appropriate beam direction requires O(log(n)).

**Algorithm 1** Proposed beamforming algorithm**Input:**$HR_{dir}^{a}\;or$ 〈$HR_{dir}^{e}$, $CSI_{AP_{CS}^{e}}$〉, ω, $\theta_{ed}$, dir1:Check ${}^{\forall }$ $inter-BSSNAV_{A}\left[i\right]$2:**if**${}^{\exists }$inter-$NAV_{A}\left[i\right]$ is set **then**3: **if** ω < $\theta_{ed}$ **then**4:  *Increase* ω5: **else**6:  dir = Result of Algorithm 17:  **if** dir is $HR_{dir}^{a}$ **then**8:   Beamform to $HR_{dir}^{a}$9:  **else**10:   Beamform with 〈$HR_{dir}^{e}$, $CSI_{AP_{CS}^{e}}\rangle$11:  **end if**12: **end if**13:**else**14: Immediately transmit DATA through $AP^{a}$15:**end if**

**Algorithm 2** Joint searching for direction and CSI**Input:**$LR_{dir}^{a}$, $HR_{dir}^{a}$, $AP_{CS}^{a}$, $CSI_{AP_{CS}^{e}}$, $HR_{dir}^{e}$, $d_{\alpha }$, $d_{\beta }$ **Output:** Appropriate direction for beamforming1:Initialize Beam search rate (BSR)2:Listen channel3:**if**$AP^{a}$ is dominant **then**4: $LR_{dir}^{a}$ = $Search_{LR}^{a}(AP_{CS}^{a}$, BSR)5: $HR_{dir}^{a}$ = $Search_{HR}^{a}(LR_{dir}^{a}$, BSR)6: return $HR_{dir}^{a}$
7:**else**8: Sort candidate $AP^{e}s$ based on signal strength9: Select dominant alien $AP^{e}$10: Set $\langle d_{\alpha },d_{\beta }\rangle$11: $HR_{dir}^{e}$ = $Search_{HR}^{e}(\langle d_{\alpha },d_{\beta }\rangle$, BSR)12: Set $CSI_{AP_{CS}^{e}}$13: return 〈$HR_{dir}^{e}$, $CSI_{AP_{CS}^{e}}$〉14:**end if**

### 4.3. Analytical Model

In this section, the total elapsed time for D/UL MU transmissions are formulated. This measurement is used for calculating the lost time of the denied transmission of STA and can be utilized for computing the expected lost throughput. The analysis regarding the expected lost throughput can be conducted with the expected lost time as red-lined in Figure 5. Furthermore, fairness on wireless medium access is also used to compare DCF with our proposed algorithm using Jain’s index [25]. The used parameters and descriptions for the mathematical analysis are summarized in Table 3.

#### 4.3.1. Total Elapsed Time of D/UL MU Transmission

STAs which communicate with AP for D/UL follow specific transmission procedure as defined in the IEEE 802.11ax TGax standardization.

In case of DL MU transmission, AP initiates IEEE 802.11ax-based transmission procedure by sending request-to-send (RTS) frame to its associated STAs. After short inter-frame space (SIFS), STAs responds with a clear-to-send (CTS) to the AP announcing that they are ready for receiving DL MU transmission data. Then, the AP concurrently transmits DL MU data to STAs. After receiving all data from the AP, STAs responds with a block acknowledgement (BACK) to the AP in order to inform of successful reception. Finally, the AP sends contention free end (CFE) to reset its NAV of STAs.

In case of UL MU transmission, STAs that want to upload data to AP send MU RTS frame to the AP. The AP responds with CTS and sends NDP/NDPA frames before transmitting *trigger* frame which initiates STAs’ UL MU transmission. After STAs trigger frame reception, the STAs concurrently transmit their data. After the termination of STAs’ data transmission procedure, the AP sends MU ACK back to STAs for informing of successful UL data transmission. Finally, it transmits CFE to STAs, same as in the DL procedure, and STAs reset the corresponding NAV.

Based on this description, the total elapsed time for a single downlink and uplink MU transmission can be formulated as (1) and (2), respectively.

(1) $$\begin{array}{ccc} T_{\mathrm{mu}}^{d}\left(U_{\mathrm{mu}},V_{s},B_{\mathrm{ru}}\right) & = & T_{\mathrm{mu}-\mathrm{RTS}}\left(U_{\mathrm{mu}}\right)+T_{\mathrm{SIFS}}+T_{\mathrm{CTS}}+T_{\mathrm{SIFS}}+T_{\mathrm{mu},d}^{\mathrm{DATA}}\left(U_{\mathrm{mu}},V_{s},B_{\mathrm{ru}}\right)+\\ & & T_{\mathrm{SIFS}}+T_{\mathrm{BA}}+\mathrm{AIFS}+T_{e} \end{array}$$

(2) $$\begin{array}{ccc} T_{\mathrm{mu}}^{u}\left(U_{\mathrm{mu}},V_{m},V_{s},B_{\mathrm{ru}}\right) & = & T_{\mathrm{mu}-\mathrm{RTS}}\left(U_{\mathrm{mu}}\right)+T_{\mathrm{SIFS}}+T_{\mathrm{CTS}}+T_{\mathrm{SIFS}}+T_{\mathrm{trigger}}\left(U_{\mathrm{mu}}\right)+\\ & & T_{\mathrm{SIFS}}+T_{\mathrm{mu},u}^{\mathrm{DATA}}\left(V_{s},B_{\mathrm{ru}}\right)+T_{\mathrm{SIFS}}+T_{\mathrm{mu}-\mathrm{ACK}}\left(V_{m}\right)+T_{e} \end{array}$$

The above two equations, i.e., (1) and (2), include the elapsed time of backoff, data transmission time, and control frames exchange times in D/UL transmission. $T_{\mathrm{mu}}^{d}\left(U_{\mathrm{mu}},V_{s},B_{\mathrm{ru}}\right)$ and $T_{\mathrm{mu}}^{u}\left(U_{\mathrm{mu}},V_{m},V_{s},B_{\mathrm{ru}}\right)$ refer to the total duration of DL and UL MU transmission respectively. They include commonly $T_{\mathrm{mu}-\mathrm{RTS}}\left(U_{\mathrm{mu}}\right)+T_{\mathrm{SIFS}}+T_{\mathrm{CTS}}+T_{\mathrm{SIFS}}$ for initial control message exchange procedure. $T_{\mathrm{mu},d}^{\mathrm{DATA}}\left(U_{\mathrm{mu}},V_{s},B_{\mathrm{ru}}\right)$ in DL MU transmission represent required time for data transmission for $U_{\mathrm{mu}}$ with $R(V_{s},B_{\mathrm{ru}})$ per each STA. After the transmission of data through DL after SIFS, STAs transmit BACK, which consumes $T_{\mathrm{BA}}$ and idle for $\mathrm{AIFS}+T_{e}$ which is the duration of arbitrary inter-symbol space (AIFS) and CFE. $T_{\mathrm{trigger}}\left(U_{\mathrm{mu}}\right)$ in UL MU transmission and $T_{\mathrm{mu},u}^{\mathrm{DATA}}\left(V_{s},B_{\mathrm{ru}}\right)$ stand for the duration of trigger frame to $U_{\mathrm{mu}}$ UL MU STAs and the required time for MU UL data transmission, respectively. Finally, after SIFS, $T_{\mathrm{mu}-\mathrm{ACK}}\left(V_{m}\right)+T_{e}$ is required for terminating the entire UL MU transmission procedures. These are utilized to calculate the cost values of the expected lost time $l_{t}$ (Section 4.3.2) and the expected lost throughput $E_{L_{Th}}$ (Section 4.3.3) in the following subsections.

#### 4.3.2. Expected Lost Time, $l_{t}$

The τ function that is defined as

(3) $$l_{t}=\tau \left(\sum_{i=0}^{N_{A}}{\mathrm{NAV}}_{i}\right)-\tau \left({\mathrm{NAV}}_{m}\right)$$

is used for calculating the total time difference depending on NAV values when intra-BSS NAV and all other inter-BSS NAVs are reset to zero. The (1) and (2) are used to calculate the time of each NAV duration. Thus, the (3) represents the delayed period of STA affected by the inter-BSS NAVs set which are generated by neighboring APs in dense deployment environments.

#### 4.3.3. Expected Throughput Loss, $E_{Th}$

Based on (3), the expected throughput loss of STA during delayed period can be calculated under missed control frames and data frames. Ahead of the detailed analysis, note that all data rates for control frames are statically set to 6 Mbps which is the data rate of a single spatial stream with 20 MHz channel in IEEE 802.11ax (i.e., R(1,20 MHz)). In addition, the *n* is proportional to $N_{A}$ where $n=E_{S}N_{A}$ and $E_{S}$ represent the average neighboring $N_{S}$. The expected loss of control and data frames can be analytically formulated as in the following; (4) and (5).

(4) $$E_{L_{c}}=\underset{T_{c}^{*}}{\underset{︸}{l_{t}\cdot \left\lceil \frac{l_{c}}{R(1,20\;\mathrm{MHz})}\right\rceil }}\cdot \underset{P_{c}^{*}}{\underset{︸}{l_{t}\cdot {\left(1-\frac{B_{r}}{l_{t}}\right)}^{\frac{n-1}{n}}\cdot {\left(\frac{B_{r}}{l_{t}}\right)}^{\frac{1}{n}}}}$$

where $E_{L_{c}}$ stands for the expected throughput loss of control frame. In (4), $T_{c}^{*}$ denotes the throughput loss during $l_{t}$ with $\left\lceil \frac{l_{c}}{R(1,20\;\mathrm{MHz})}\right\rceil$ size of control frame per unit time and $P_{c}^{*}$ stands for the successful probability to find the appropriate beamformee with BSR $B_{r}$ in time period $l_{t}$ in *n* counts.

Similarly, the expected throughput loss of data frame, i.e., $E_{L_{d}}$, can be formulated as follows:

(5) $$E_{L_{d}}=\underset{T_{d}^{*}}{\underset{︸}{l_{t}\cdot \left\lceil \frac{l_{d}}{R(V_{s},B_{\mathrm{ru}})}\right\rceil }}\cdot \underset{P_{d}^{*}}{\underset{︸}{l_{d}\cdot {\left(1-\frac{B_{r}}{l_{t}}\right)}^{\frac{n-1}{n}}\cdot {\left(\frac{B_{r}}{l_{t}}\right)}^{\frac{1}{n}}}}$$

where $T_{d}^{*}$ and $P_{d}^{*}$ denote the throughput loss duration $l_{t}$ with data rate $R(V_{s},B_{ru})$ and the probability for successfully finding the appropriate beamformee with BSR $B_{r}$ for the duration $l_{t}$ in *n* counts, respectively. Finally, the total expected loss of throughput can be denoted by:

(6) $$E_{L_{Th}}=E_{L_{c}}+E_{L_{d}}.$$

#### 4.3.4. Jain’s Index J for Fairness

In addition, the medium access fairness can be evaluated by the following Jain’s index, i.e.,

(7) $$J\left(x_{1}^{t},x_{2}^{t},\cdots ,x_{i}^{t}\right)≜\frac{{\left(\sum_{i=1}^{N_{S}}x_{i}^{t}\right)}^{2}}{N_{S}\cdot \sum_{i=1}^{N_{S}}{\left(x_{i}^{t}\right)}^{2}}$$

where each of $x_{i}^{t}$ represents throughput of i−th STA in time step *t*.

## 5. Performance Evaluation

In this section, we discuss the simulation setting and results regarding the delay scenario which is considered in the previous section. We simulate the denied transmission period based on the analytic model which is presented in the previous section for both U/DL MU transmission. In addition, we investigate throughput gain of U/DL MU transmission for estimated time loss, which is caused by the interference, based on (4), (5), and (6). The proposed medium access scheme shows that it enables STAs which are located in densely deployed IoT environment to communicate via MU transmission with mitigated interference. Consequently, the performance in terms of the expected amount of transceived data of STAs is improved with the proposed scheme. Finally, we evaluate our proposed medium access control protocol in terms of fairness using Jain’s index. We compare the fairness values of the ordinary DCF and the proposed algorithm under different options and various numbers of STAs. Note that all simulation environments assumed that the channel condition between STAs and APs is stationary.

### 5.1. Simulation Setting and Overview

Our simulation is designed and implemented based on the assumptions and formulations in Section 4 and Table 3, in order to precisely operate with obeying these settings. The parameters and corresponding values used in our simulation are summarized in Table 4. The simulation environment is constructed as follows: there are 8 APs and STAs from 4 to 64 which are located in densely deployed OBSSs grid topology. The STAs are assumed to always have data to transmit in their transmission queue to the associated AP. Thus, the STAs, which exist in the OBSSs area, severely compete for spectrum resources so that each of them affects neighboring STAs with respect to the delay and throughput gain performance. Consequently, the STAs within the OBSSs are delayed to transmit data and their throughput gain is deteriorated due to their set of multiple inter-BSS NAVs and intra-BSS NAV. For simulation variables, we assumed that channel state is stationary and the positions of APs and STAs are fixed, which means no random simulation variables exist. In addition, the simulations are conducted with the aforementioned environment settings and simulation parameters in Table 4, and by varying the number of the existing STAs in the topology, MCS, and BSR in DL and UL scenario respectively. Note that each of the simulation results is derived from multiple independent experiments with predefined fixed simulation parameters.

In this simulation environment, we specifically designed a delay scenario where the inter-BSS NAVs of specific target STA is successively set due to neighboring APs which successively initiate transmission with their associated STAs. Thus, the STA is delayed because of successive sets of inter-BSS NAVs, and has to wait until all of them are reset. The delay duration of the STA can be minimized to nearly wait time threshold $\theta_{ed}$ in Algorithm 2 by taking advantage of our proposed algorithms above. The total bandwidth of a carrier is 160MHz and FFT size is adopted as 256. In addition, the aggregated medium access control protocol data unit (A-MPDU) consists of $P_{a}$ packets with symbol duration ρ (16 μs), which is used for transmission simulations. The size of $l_{c}$, including ACK, SIFS, and other control frames, is set to 123 Bytes and $l_{d}$ is 1460Bytes. In addition, $P_{a}$ frames in the size of $l_{d}$ are aggregated as an A-MPDU for simulations. Moreover, the AP with 8 antennas serves STAs that each of them is equipped with 2 antennas for enabling transmission through multiple spatial streams. First, we simulate the time loss in U/DL transmission under hyper-dense deployment scenarios by varying modulation and coding scheme (MCS) levels and the number of STAs which delay an STA such as $STA_{2}$ in Figure 5. Furthermore, dense constellations which support up to 32 STAs are considered to evaluate the stability of time loss for the delayed STA. In addition, we simulate the expected throughput gain of STAs regarding U/DL transmission with various parameters for the STAs, MCS, and beam search rate (BSR). Finally, the fairness of the medium access for each STA is compared with DCF by calculating the Jain’s index. Simulation results show that the fairness of the proposed medium access scheme outperforms the DCF.

### 5.2. Simulation Results and Discussions

**Figure 7:**
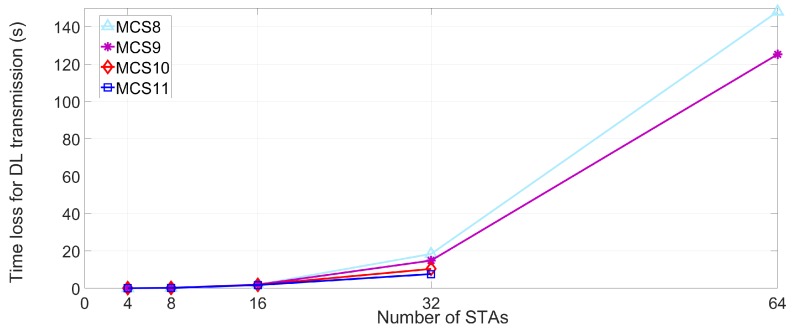
Expected time loss $l_{t}$ for DL transmission.

**Figure 8:**
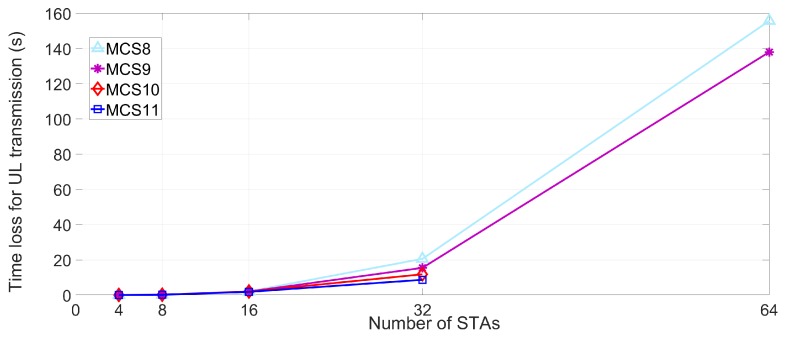
Expected time loss $l_{t}$ for UL transmission.

**Figure 9:**
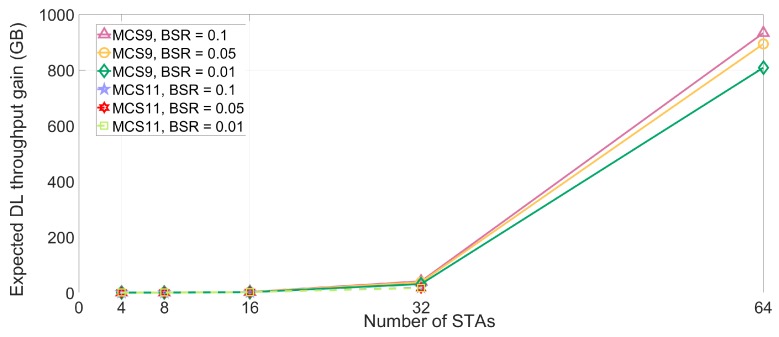
Expected throughput gain for DL transmission.

**Figure 10:**
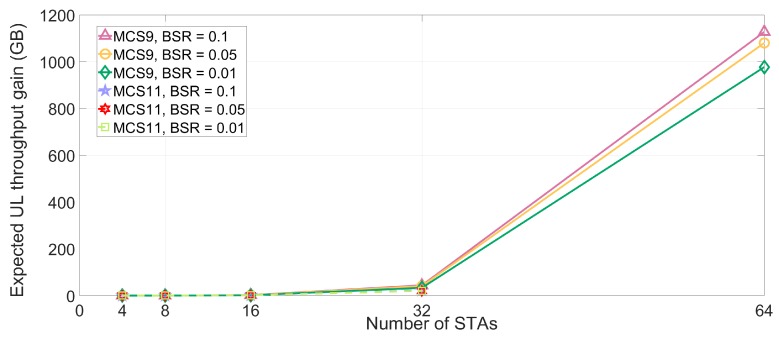
Expected throughput gain for UL transmission.

The $l_{t}$ values of DL and UL MU transmission are simulated in dense deployment environments under the various MCS and STAs numbers, as presented in Figure 7 and Figure 8. Based on (3), the time loss of STA is measured using the parameters in Table 4. The time loss of STA is sharply increased as the number of neighboring STAs increases. As the number of the interfering STAs reaches 64, the STA which uses MCS9 and MCS10 cannot transmit any data in effect. The reason for this phenomenon is the multi-NAVs policy of IEEE 802.11ax where the inter-BSS NAVs are increased to manage the possible congestion for STA. Furthermore, the inter-BSS NAVs are proportional to the number of neighboring or interfering APs and associated STAs. Consequently, the increased set intra-BSS NAVs and inter-BSS NAVs elongate the expected time loss of STA, which is located in densely deployed IoT environment, and deteriorate the QoS of STA due to the delay issue. Figure 9 and Figure 10 show the expected throughput gain of D/UL MU transmission based on (3), (4), and (5) varying BSR, MCS level, and the number of interfering STAs. Note that the physical data rate values for calculating the throughput with various numbers of STAs and MCS level are referenced in [17]. The elongated delay issue caused by the neighboring STAs results in time loss for data transmission and reception of STA as shown in Figure 7 and Figure 8. The delay issue is effectively handled by the proposed beam alignment algorithm, which finds alternative AP (if associated AP is saturated), or appropriate beamforming direction (associated AP is available with different beamforming direction). After finding the appropriate alien AP or alternative beam direction for associated AP, STA with our algorithm can resume its operation of Tx and Rx. The results of throughput gain simulation show that the proposed algorithm is more effective in densely deployed scenario where 64 STAs affect an STA within a BSS coverage than other scenarios (4, 8, 16, 32 STAs). Thus, the performance of our algorithm for mitigating the interference is effective for both general and densely deployed IoT scenarios.

**Figure 11:**
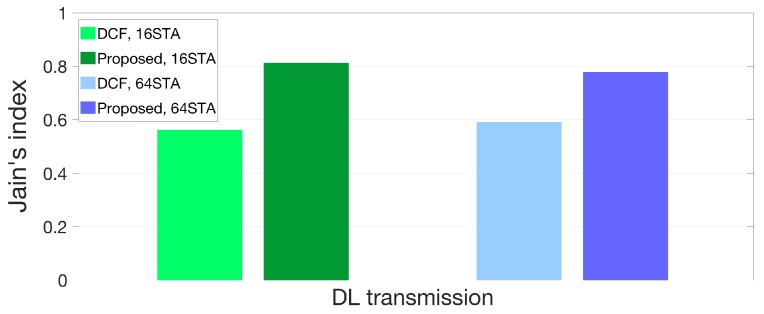
Fairness comparison between DCF and proposed protocol in DL scenario.

**Figure 12:**
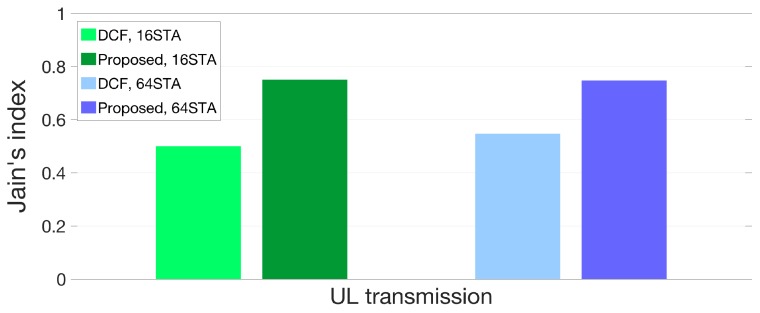
Fairness comparison between DCF and proposed protocol in UL scenario.

Finally, Figure 11 and Figure 12 represent a comparison of the fairness index values for the medium access between DCF and our proposed algorithm. The results are analyzed with (7) and throughput gain in Figure 9 and Figure 10. The fairness of our proposed algorithm is improved to 44% and 31% compared to DCF when the numbers of STAs are 16 and 64 respectively in DL transmission as shown in Figure 11. In case of UL transmission, the fairness of the medium access is improved up to 50% and 36% when the numbers of interfering STAs are 16 and 64 respectively as Figure 12. In conclusion, our proposed algorithm enhances not only throughput gain of each STA but the fairness of the channel access efficiency in hyper-densely deployed environments. The STA with our algorithm does not act selfishly but altruistically with the wait threshold so that STAs in a BSS can operate cooperatively.

## 6. Conclusions Remarks and Future Work

In this paper, we demonstrated the possible delay problem of IEEE 802.11ax in a hyper-dense deployment of IoT wireless devices due to multiple inter-BSS NAVs settings and analyzed the expected lost time and throughput of victim STAs. For addressing this issue, we firstly propose a swift and low-complexity beam direction selection algorithm based on an opportunistic beamforming for IEEE 802.11ax-based IoT systems in terms of the joint optimization of delay reduction and interference mitigation. In addition, we evaluated the performance of our proposed algorithm in terms of the throughput and fairness, and clearly show that our proposed algorithm can relieve delay issue and fairness of STAs. As future work, performance of proposed interference mitigating algorithm will be compared to previously proposed medium access algorithms. In addition, our proposed algorithm will be improved for accounting the dynamics of channel condition (including the measurements of CSI), i.e., miscellaneous traffic in non-saturation case, for realistic applications for densely deployed 802.11ax-based IoT environments.

## Figures and Tables

**Table 1:** A comparison between the IEEE 802.11ac and the IEEE 802.11ax.

	IEEE 802.11ac	IEEE 802.11ax
Band (GHz)	5	2.4 and 5
Channel bandwidth (MHz)	20, 40, 80, 80 + 80, 160	20, 40, 80, 80 + 80, 160
Modulation	BPSK, QPSK, 16QAM, 64QAM, 256QAM	1024QAM is newly added
FFT size	64, 128, 256, 512	256, 512, 1024, 2048
Subcarrier spacing (KHz)	312.5	78.12
Symbol duration (us)	3.2	12.8
CP (us)	0.4 and 0.8	0.8, 1.6, and 3.2
FEC	BCC, LDPC (optional)	LDPC
Spatial stream (SS)	Up to 8 SS for each AP	Up to 8 SS for each AP
	Up to 4 SS for each STA	Up to 4 SS for each STA
MU-MIMO	DL MU-MIMO	UL/DL MU-MIMO

**Table 2:** Comparison with other OFDMA protocols.

	Proposed	[21]	[22]	[23]	[24]
MU transmission	✓	✓	✓	✓	✓
MU access	✓	✓	✓	✓	✓
MU diversity	✓		✓		
Simple signal exchange	✓	✓	✓	✓	
CSI measurement	✓				

**Table 3:** Parameters used in analysis.

Parameter	Description
$N_{A}$	Number of APs
$N_{S}$	Number of STAs
$U_{\mathrm{mu}}$	Number of MU STAs
$\tau \left(x\right)$	Return time when x=0
NAV${}_{i}$	*i*-th inter-BSS NAV
NAV${}_{m}$	intra-BSS NAV
$L_{c}$	Length of control frame
$L_{d}$	Length of data frame
$B_{r}$	Beamforming search rate
$x_{i}^{t}$	Throughput of *i*-th STA in time *t*
*n*	Required count to find appropriate beamformees
$V_{s}$	Number of SU-MIMO spatial streams per STA
$V_{m}$	Number of MU-MIMO spatial streams per STA
$R(V_{s},B_{\mathrm{ru}})$	Data rate
$B_{\mathrm{ru}}$	Bandwidth of RU
$L_{D}$	Packet size
$P_{a}$	Number of aggregated packets in A-MPDU

**Table 4:** Simulation parameters.

Parameter	Description
*B*	160 MHz
FFT	256
$L_{d}$	1460 bytes
$P_{a}$	256
$CW_{\min}$	32
$CW_{\max}$	1024
SIFS	16 μs
aSlotTime	9 μs
ρ	16 μs
$N_{A}$	8
$N_{S}$	from 8 up to 64
